# Experimental Study on Microstructure and Erosion Mechanisms of Solid Waste Cemented Paste Backfill under the Combined Action of Dry–Wet Cycles and Sulphate Erosion

**DOI:** 10.3390/ma15041484

**Published:** 2022-02-16

**Authors:** Kexin Li, Xilin Li, Chuanyang Du, Haowen Xue, Qi Sun, Ling Liu

**Affiliations:** 1 School of Civil Engineering, Liaoning Technical University, Fuxin 123000, China; likexin202104@163.com (K.L.); d18342847181@163.com (C.D.); xhw02261112@163.com (H.X.); liuling@lntu.edu.cn (L.L.); 2 School of Architecture and Transportation, Liaoning Technical University, Fuxin 123000, China; sunqi@lntu.edu.cn

**Keywords:** solid waste cemented paste backfill, sulphate erosion, microstructure, erosion mechanism

## Abstract

Solid waste cemented paste backfill (SWCPB) meets the needs of coal mining area management. SWCPB is a cementitious paste backfill material without added cement and is made only from oil shale residue (OSR), steel slag (SS), soda residue (SR) and water. In this study, mine water characteristics were simulated by combining dry–wet cycling experiments with sulphate erosion experiments. SWCPB was assessed regarding appearance, mass loss, and unconfined compressive strength (UCS), and the erosion products were microscopically analysed with X-ray diffraction (XRD), scanning electron microscopy (SEM) and energy dispersive spectroscopy (EDS). The mechanism for erosion of the SWCPB by sulphate-rich mine water was comprehensively analysed and revealed. Research showed that the erosion mechanism was divided into two parts: chemical and physical erosion. Low concentrations of sodium sulphate promoted hydration, thereby contributing to the increased mass and strength of SWCPB. At high sodium sulphate concentrations, the erosion mainly consumed Ca(OH)_2_ within the material, and the main generated erosion products were gypsum and ettringite (AFt). This was accompanied by the destructive effects of Na_2_SO_4_ crystal expansion, which resulted in damage and the reduced workability of the SWCPB. The whole erosion process was continuous, mainly due to transformations of pits, pores and cracks. The conclusions of this study may provide appropriate guidance for application of SWCPB materials in the treatment of coal mine backfills. In addition, the corresponding theoretical analysis of the erosion mechanism for SWCPB materials is provided.

## 1. Introduction

China is very rich in coal resources, and the scale and intensity of current coal mining is at an unprecedented level [1]. Extensive mining has caused a scarcity of coal resources, as well as damage to the ecological environment. To effectively protect and utilise resources and reduce ecological pollution, backfilling mining is the best option [2]. The best filling method is cemented paste backfill (CPB). As one of the core CPB technologies, backfill materials have received much attention from experts and scholars. Current research in backfilling materials is focused on developing towards low cost, high strength, easy delivery, easy production, and simple processes [3]. Solid waste cemented paste backfill (SWCPB) material uses synergistic stimulation between solid wastes and is made entirely of solid waste mixed with water. Song et al [4]. prepared a ternary blend using SS-fly ash (FA)-ground granulated blast furnace slag (GGBS). The SS–GGBS system was found to contribute to the overall strength of the cemented material. Li et al [5]. used SS–OSR–GGBS as the raw material to prepare the filling slurry, and the mixing ratio was optimised with a combination of response surface and multi-objective methods. The prepared material meets filling requirements and is inexpensive, with low environmental impact on groundwater. These studies have effectively contributed to the development of SWCPB. Compared to other CPB materials, SWCPB requires no chemical excitation and no cement addition, thus enabling the 100% application of solid waste. It has the advantages of a wide range of raw materials, low cost and low environmental pollution. It has a wide range of applications and development prospects.

When using CPB for underground backfill in coal mines, the complex mine water and filling environments have significant impacts on the performance of CPB [6]. According to relevant statistics, most mine water in mining areas contains sulphates, and the proportion of high sulphate mines (mine water with a sulphate content greater than 250 mg/L) in a typical mine survey is 72.5% [7]. CPB exposed to a high sulphate environment for a long time will crack due to sulphate erosion. In the long run, this affects the filling effect and eventually leads to top slab subsidence in the filling area, which consequently causes a geological disaster [8]. Therefore, this cannot be ignored during the development of new materials. Many scholars have studied various previously developed filling materials for sulphate attack environments. Li et al [9]. found that the sulphate environment and self-drying affected the early strength of the filling material in a study of filling pastes. Moreover, the greater the concentration of sulphate is in the environment, the more pronounced its effect on early strength. Yin and Yan et al [10,11]. studied the effect of sulphate erosion on CPB and found that the sulphate concentration influenced the evolution of strain and the free expansion ratio of CPB. Wang et al [12]. modelled the effects of mine water environments in metal mines by varying the concentration of sulphate. A BP neural network prediction model for the UCS of CPB was also developed. The results showed that sulphate concentration had a strong impact on the formation and crystallisation of hydration products, which directly affected the pre-failure and post-failure behaviour and bulk volume of CPB at the macroscale. Fall and Pokharel [13,14] investigated the combined effects of sulphate and temperature on CPB. The effect of the coupling action on strength development by the CPB and the saturation hydraulic conductivity was investigated. The magnitude of the effect was found to depend on the values of the initial sulphate content and the curing temperature. Dong et al [15]. investigated the effect of sulphide on the long-term strength of lead–zinc tailing sand cemented backfill by preparing CPB specimens with different concentrations of sulphide. The differences between internal sulphide and external sulphate attacks were also compared. Aldhafeeri et al [16]. investigated the effect of the initial sulphate content on the reactivity of cemented paste tailing (CPT) by performing oxygen consumption (OC) tests. It was found that the reactivities of the CPT specimens increased with increasing sulphate content. Increasing the cement content or replacing cement with mineral admixtures reduced the reactivity of the slurry. Changes in runoff due to mining activities or rainfall caused the level of the mine water to fluctuate up and down. This placed the CPB in a dry–wet cycle in the working area [17]. In this regard, Chen et al [18]. investigated cemented coal gangue–fly ash backfill (CGFB) and erosion by an Na_2_SO_4_ solution during dry–wet cycling. The results showed that the driving force for the incorporation of sulphate ions into the CGFB sample was greatest for immersion in a 15% Na_2_SO_4_ solution, and the mass and strength increased the fastest. Moreover, the content of the eroding ions was decreasingly distributed from the outside to the inside.

In addition, differences in the type and structure of products formed during sulphate erosion of different backfill materials resulted in different erosion mechanisms. Cui et al [19,20]. found that the effect of sulphate on the strength of fly ash cemented paste backfill (FCPB) was largely dependent on the hydration products formed from FCPB after different soaking times. The presence of AFt improved the denseness in the early stages of erosion but produced greater erosion damage in the later stages. CPB with an appropriate admixture of fly ash improved the internal structure of FCPB and increased resistance to sulphate erosion. Bellmann et al [21]. compared the effects of gypsum on the performance of CPB in erosion products by means of thermodynamic calculations based on indoor experiments and field tests. They found that the effect of gypsum on the cemented backfill material used in the field tests was less than the effect of gypsum generated by sulphate erosion in the laboratory. Zheng et al [22]. also found that resistance to sulphate erosion significantly varied for different geopolymers. When the hydration product was a structurally stable sodium aluminium silicate (N–A–S–H) gel, the sulphate erosion process was dominated by the influence of the eroding cations on the product structure; when the hydration product was a calcium aluminosilicate hydrate (C–A–S–H) gel, the sulphate erosion mechanism was similar to that of ordinary Portland cement (OPC).

Scholars have performed in-depth studies an applications of different types of filling materials. The focus has been on the use of cement-based materials, alkali-activated materials and partially solid waste-added materials. Less research has been carried out on materials that are cement-free and additive-free and where solid waste acts synergistically to generate cementitious strength. In particular, much less has been reported on the damage patterns and mechanisms of SWCPB materials attacked by sulphates. SWCPB is a material made entirely from a mixture of solid waste without the addition of cement, and its destruction mechanism may be different from that of conventional CPB. For this paper, variations in SWCPB performance under the effects of different sulphate concentrations and dry–wet cycles were explored through macroscopic tests such as appearance, mass loss and strength tests. The mechanism for the erosion of SWCPB during dry and wet sulphate cycles was analysed with XRD, SEM and EDS tests. Theoretical support was provided for studies of SWCPB performance in a sulphate mine water environment.

## 2. Materials and Methods

### 2.1. Materials

**Figure 1:**
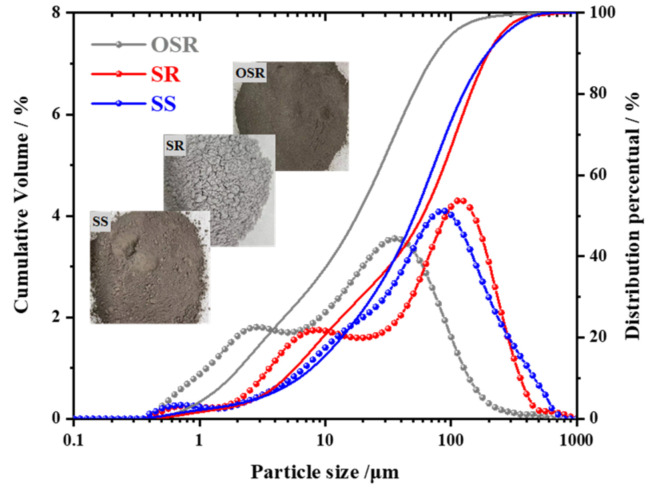
Particle size distribution curves.

SWCPB, which is a paste material with a high concentration and fluidity, was prepared by mixing oil shale residue (OSR) and steel slag (SS) with a small amount of soda residue (SR) and water. The OSR used in this study was solid waste from the West Open Pit Coal Mine in Fushun (Fushun, China). The material was a coal-associated solid waste, it was crushed and used with a fineness of 24%, its particles exhibited sizes largely concentrated in the range 0.01–0.1 mm, and the specific surface area was 1.190 m^2^/g. SS, with a crushed specific surface area of 0.497 m^2^/g, was taken from the solid waste of Anshan Iron and Steel Group (Anshan, China), and the particle sizes were equal to approximately 0.1 mm. SR was obtained from Tangshan Sanyou Chemical Group (Tangshan, China). The specific surface area after grinding was 0.54 m^2^/g, and the particle sizes were mostly concentrated at 0.1–0.15 mm. The particle size distribution curve for the raw material was obtained using a laser particle size meter, as shown in Figure 1. The chemical composition is shown in Table 1.

Based on previous findings of our subject group [23], the flowability and strength of the SWCPB material were taken into consideration. The optimum mixing ratios and test data for SWCPB were obtained after optimisation, as shown in Table 2. The paste was prepared by mixing the raw materials at the optimum mix ratio. The fresh slurry was cast in Ф50 × 100 mm cylindrical moulds, demoulded after 24 h and tested after standard curing to the appropriate age. The pH of the SWCPB leachate was 11.21, which is alkaline.

### 2.2. Methods

The test specimens for sulphate and wet/dry cycle tests were started after 26 d of curing and mainly carried out with reference to the sulphate attack resistance test in the Chinese Standard for Test Methods of Long-term Performance and Durability of Ordinary Concrete (GBT50082-2009 Beijing, China) [24]. To accelerate the deterioration caused by sulphate erosion, drying and warming were used to carry out alternate dry–wet tests. In this study, oven drying and natural immersion at room temperature were used to simulate alternating dry–wet conditions.

Configuring the solutions: Analytically pure Na_2_SO_4_ crystals were used for solution preparation. Due to the low solubility of Na_2_SO_4_, the solution was prepared with warm water after weighing. The solution was continuously stirred with a glass rod to prevent the formation of a precipitate. The erosion solution for this test was a Na_2_SO_4_ solution, the mass concentrations were set at 5% and 10%, and clear water erosion was used in control experiments.

Test procedure: Ten groups of 3 specimens were used for each solution concentration. The specimens were soaked in a salt solution for 10 h, removed and dried on the surface, left to stand at room temperature for 2 h and put into a drying oven for 10 h, removed and weighed, and cooled at room temperature for 2 h. This cycle took 24 h. Every 2 cycles (48 h) were a test cycle until the UCS test results of the specimens could not be compared, and then the cycle tests were stopped.

**Figure 2:**
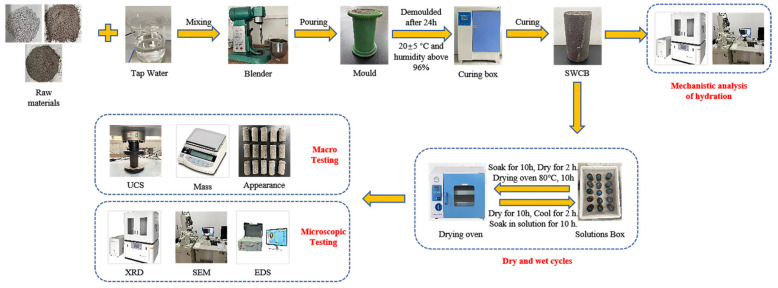
Experimental flow diagram.

The experimental procedure is shown in Figure 2.

The changes in appearance, UCS and mass of the specimens were observed after different cycles, and statistical comparisons were recorded. The mass change considered was the mass loss rate Δ*m*_Ti_ (%), which was calculated with Equation (1): the difference between the mass *m*_0i_ of the ith specimen in the initial state and the mass *m*_Ti_ after the Tth cycle was recorded as the mass loss of the ith specimen. The ratio of the mass loss (Δ*m*_Ti_) to the initial mass (*m*_0i_) was the mass loss rate. When the cumulative mass loss due to dry–wet cycles was <2%, it was taken to reflect the resistance of the SWCPB to dry–wet cycles [25].

(1) $$\Delta m_{\mathrm{Ti}}=\frac{m_{0i}-m_{\mathrm{Ti}}}{m_{0i}}\times 100\%$$

The specimens were also subjected to SEM and XRD microscopic characterisation studies at the middle and late stages of testing. The crystal structure and composition of SWCPB were examined with X-ray diffraction (XRD) on a XRD-6100 X-ray diffractometer (Shimadzu, Kyoto, Japan) with Cu-Kα radiation (λ = 1.54056Å) at a rate of 4°/min in the range of 5°–80°. XRD analysis was carried out using MDI Jade 6.0 software. A JSM-7500F scanning electron microscope (JEOL, Tokyo, Japan) was used to analyse microstructural changes. A FYFS-2002E energy dispersive spectroscopy (EDS) detector (Fangyuan Instrument, Wuhan, China) was used for the elemental analyses of erosion products.

## 3. Results

It is commonly believed that the erosion resistance of a material is related to its own properties. Since no cement or chemical reagents were added to the SWCPB, it relies on synergistic hydration between the component materials to develop strength. Therefore, the analysis of the erosion mechanism for SWCPB under sulphate attack must be preceded by an analysis of synergistic hydration involving the solid wastes. The results of the erosion experiments are then be analysed and discussed on the basis of hydration.

### 3.1. Synergies among Solid Wastes

**Figure 3:**
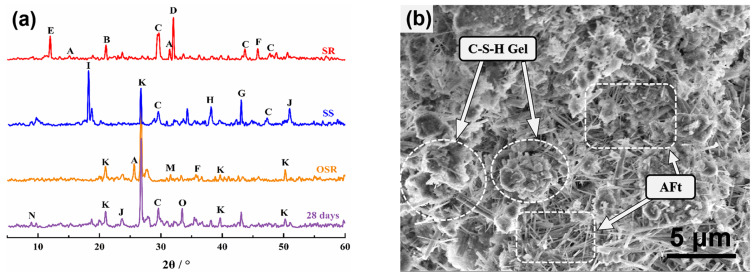
Microscopic testing of specimens: (**a**) XRD of raw material compared to SWCPB; (**b**) SEM of SWCPB. (In (**a**): A: CaSO_4_; B: CaSO_4_·2H_2_O; C: cancrinite; D: Na_2_SO_4_; E: magnesian calcite; F: NaCl; G: RO; H: C_2_S and C_3_S; I: calcium ferric aluminate; J: calcium hydroxide (Ca(OH)_2_); K: quartz (SiO_2_); L: Fe_2_O_3_; M: Al_2_O_3_; N: ettringite (AFt); O: C–S–H gel).

In the course of our research on SWCPB materials, we carried out XRD and SEM tests on the raw materials and the material cured for 28 d in the standard state to analyse the reasons for strength development without the addition of cement and chemicals. The synergistic hydration mechanism was investigated as a basis for mechanistic analyses of SWCPB undergoing sulphate attack. XRD and SEM images are shown in Figure 3.

**Figure 4:**
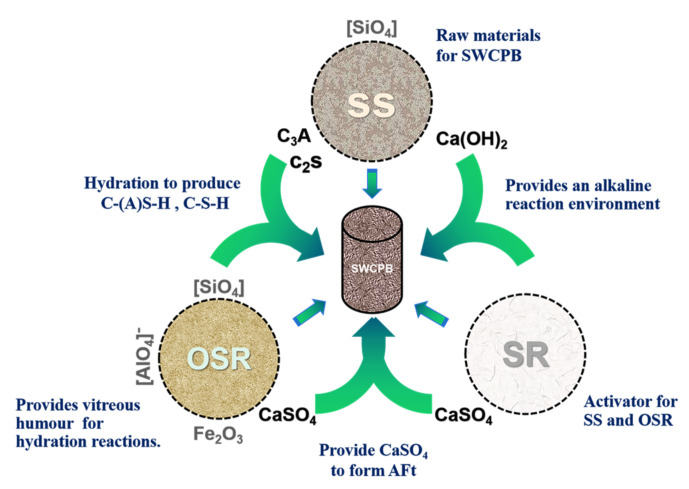
The synergism of the SWCPB.

The analysis in Figure 3a clearly shows that quartz (SiO_2_ PDF#85-0798) in the SWCPB was mainly derived from raw materials. In contrast, C–S–H gels (PDF#03-0594) and AFt (PDF#41-0217) were generated by the hydration reaction. The SEM diagram (Figure 3b) shows that at 28 d, pin-stick-like ettringite (AFt) and flocculent C–S–H gel appeared inside the specimen. Solid waste cemented paste backfill was characterised by making full use of the characteristics of raw material components, which complement each other to produce curing cementation. The synergism of the SWCPB is shown in Figure 4.

The presence of calcium hydroxide (Ca(OH)_2_) in the SS raw material, together with the SR, created an alkaline environment for the hydration reaction. This effectively stimulated the activities of SS and OSR. During this process, the OSR provided large amounts of Al_2_O_3_ and SiO_2_ for the internal hydration of the SWCPB and promoted the formation of C–S–H/C–A–S–H gels in the material. In addition, CaSO_4_ in SR and OSR was involved in the hydration reaction and produced AFt. The chemical reactions, Equations (2) and (3), for hydration occurred as follows. The interlocked combination of C–S–H gel and AFt created the cementing strength of the material. The hydration of C_2_S, C_3_S, and C_3_A in SS also increased the compressive strength of the material. Therefore, through complementary synergy between the raw materials, a hydration reaction similar to that of cement resulted.


(2) $$2\mathrm{CaO}\cdot {\mathrm{SiO}}_{2}+H_{2}O\to 2\mathrm{CaO}\cdot {\mathrm{SiO}}_{2}\cdot H_{2}O$$

(3) $$C_{3}A+3({\mathrm{CaSO}}_{4}\cdot {2H}_{2}O)+{2\mathrm{Ca}(\mathrm{OH})}_{2}+24H_{2}O\to 3\mathrm{CaO}\cdot {\mathrm{Al}}_{2}O_{3}\cdot {3\mathrm{CaSO}}_{4}\cdot 32H_{2}O$$

### 3.2. Analysis of Erosion Experiments

To investigate the evolution of work performance during the cycled wet and dry conditions for sulphate erosion, the mass loss, appearance and UCS of the SWCPB were recorded and analysed. Microscopic tests were also carried out on specimens with different erosion cycles to analyse the mechanism of erosion in conjunction with macroscopic experimental phenomena.

#### 3.2.1. Evolution of the Appearance

**Figure 5:**
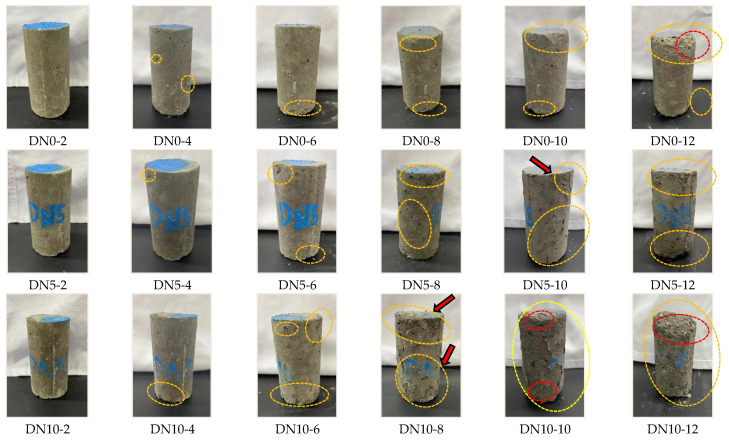
The appearance changes of different concentrations of sulphate during dry–wet erosion.

Changes in the appearance of specimens with increasing cycles and different Na_2_SO_4_ concentrations are shown in Figure 5. (Comparison photographs were recorded after drying during each dry–wet cycle. DN0 represents water erosion, DN5 represents erosion with the 5% Na_2_SO_4_ solution, and DN10 represents erosion with the 10% Na_2_SO_4_ solution).

As shown in Figure 5, SWCPB was subjected to corrosion by Na_2_SO_4_ solutions with dry–wet cycles, and the specimens mainly exhibited appearance changes during the following stages.

SWCPB specimens at 0–4 cycles (pre-erosion): There were no significant changes apparent within 0–2 cycles, and the shapes were intact and showed only a few small pits. Under the alternating effects of solution erosion and dry–wet cycles, fine surface damage began to occur at four cycles. The specimens were more heavily pitted by water erosion than they were after two cycles, and the surface skins of the specimens showed slight flaking. In contrast, the DN5 and DN10 specimens showed fewer apparent changes. The sulphate in the solution was presumed to be involved in hydration, which was considered in tandem with the results of the UCS tests and the microscopic tests.

SWCPB specimens exhibited increasing solution erosion at 4–8 cycles (mid-erosion): Small cracks appeared locally at the edges of the cylinder surface and both bottom surfaces of the specimens, as shown in Figure 5. Compared with that of DN0, the slight warping of the DN10 epidermis caused flaky peeling that then gradually increased. This resulted in the appearance of exposed internal material, such as the area shown by the arrow in Figure 5 (DN10-8).

SWCPB specimens at 8–12 cycles (post-erosion), as shown in Figure 5: Due to the erosion caused by the highly concentrated Na_2_SO_4_ solution, the surface skin of the cylinder gradually peeled off completely, and the inner layer of the material was exposed. The cracks on the surface of the specimen increased in number and gradually deepened, and the surface of the specimen exhibited a bumpy feeling. The upper and lower bottom surfaces of the specimen significantly flaked off, and the skin and inner layer of the specimen severely fell off at the corners. The specimen was severely attacked by sulphate, as shown in Figure 5 (DN10-12).

**Figure 6:**
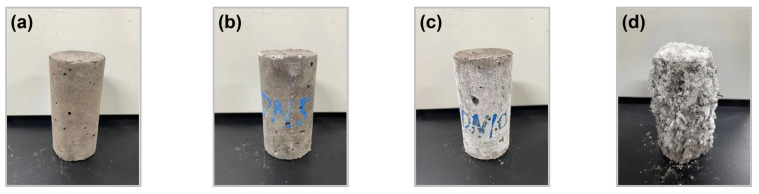
Crystalline precipitates of specimens formed after standing for 2 h at room temperature in solutions of different concentrations: (**a**) DN0; (**b**) DN5; (**c**) DN10. The crystalline precipitation of the specimens occurred during storage at the end of the experiment: (**d**) DN10.

It is worth noting that during the sulphate immersion cycles, white sulphate powder gradually appeared on the surface of the specimen after the evaporation of water during the 2 h period of drying at room temperature. More crystalline precipitation occurred on the surface of DN10, and the crystals are shown in Figure 6b,c. This crystallisation mainly occurred at the locations of small holes in the surface of the specimen or at surface cracks. For specimens stored at room temperature after cyclic compressive strength testing, more crystalline precipitate was formed, as shown in Figure 6d.

The reason for this situation is that the concentration of Na_2_SO_4_ in the specimen gradually increased during the evaporation of the water inside the specimen during in the resting stage. When the solution was supersaturated, Na_2_SO_4_ crystals precipitated. The volume expansion caused by this crystallisation process could impact the durability of the SWCPB.

#### 3.2.2. Evolution of the Mass

**Figure 7:**
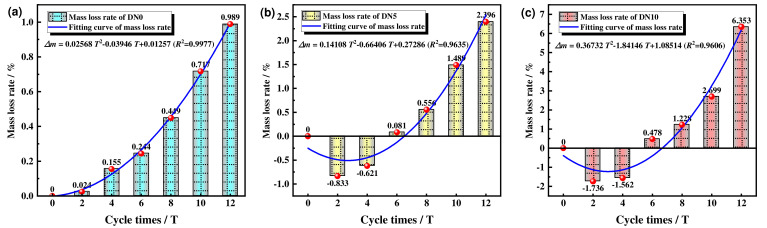
Mass losses rate of SWCPB for different cycles. (**a**) Mass loss rate and fitted curves for DN0; (**b**) Mass loss rate and fitted curves for DN5; (**c**) Mass loss rate and fitted curves for DN10.

Changes in masses were recorded during the dry cycles for specimens treated with different solutions and concentrations. Mass changes (including mass loss and average mass loss) were calculated for different solution concentrations and different numbers of cycles. The mass loss rate was calculated from Equation (1), and the relevant obtained data are shown in Figure 7.

The mass loss rates for SWCPB treated with different Na_2_SO_4_ solution erosion conditions were derived from the data and graphs, and they revealed the laws for mass change. As shown in Figure 7a, the mass loss rate of SWCPB subjected to aqueous erosion exponentially and nonlinearly increased. Figure 7b,c shows that the mass losses of the specimens subjected to sodium sulphate erosion first decreased and then increased exponentially. The trends for SWCPB mass losses for different erosion conditions are given in Equations (4)–(6), where ∆*m* is the mass loss rate and *T* is the number of cycles. The *R*^2^ values for the correlation coefficients were 0.9977, 0.9635, and 0.9606, respectively (all greater than 0.95). This indicates that the models were fitted with high accuracy and were statistically significant.

(4) $$\Delta m=0.02568T^{2}-0.03946T+0.01257\;(R^{2}=0.9977)$$

(5) $$\Delta m=0.14108T^{2}-0.66406T+0.27286\;(R^{2}=0.9635)$$

(6) $$\Delta m=0.36763T^{2}-1.84146T+1.08514\;(R^{2}=0.9606)$$

In addition to the overall comparison in Figure 7a–c, the mass loss rates for the aqueous solution erosion cycles were all greater than 0%, indicating that the mass of the specimen was consistently reduced during the erosion process. In contrast, the mass of the specimen increased and then decreased during erosion cycles with the Na_2_SO_4_ solution. This was likely caused by the initial entry of ions from the erosion solution into pores on the surface of the specimen and chemical reactions with the material inside the specimen. The insoluble material produced by the reaction adhered to the interiors of the pores, resulting in a reduction in the overall porosity of the specimen, an increase in compactness, and therefore an increase in total mass. When the concentration of the solution inside the specimen reached a certain limit, the original pores of the specimen were filled with insoluble material. At this point, as the number of cycles and the erosion of the solution increased, the excess insoluble material accumulated and caused the specimen to swell, crack, and form small fissures. This led to gradual increases in the sizes of the existing small pores and made it easier for the aggressive ions in the solution to enter and erode the interior. As a result, the compactness slowly decreased and the total mass fell sharply. Therefore, it appears that the greater the concentration of the sulphate solution was, the faster the negative growth and the greater the amount of mass change.

Comprehensive analysis showed that SWCPB had a mass loss rate of only 0.989% during test cycles for aqueous solution erosion. The mass loss rate was <2% after 10 dry–wet erosion cycles with the 5% Na_2_SO_4_ solution. These results also showed that the SWCPB material prepared from oil shale slag–steel slag–soda residue had strong resistance to dry–wet cycles and a certain degree of resistance to sulphate attack.

#### 3.2.3. Evolution of the UCS

**Figure 8:**
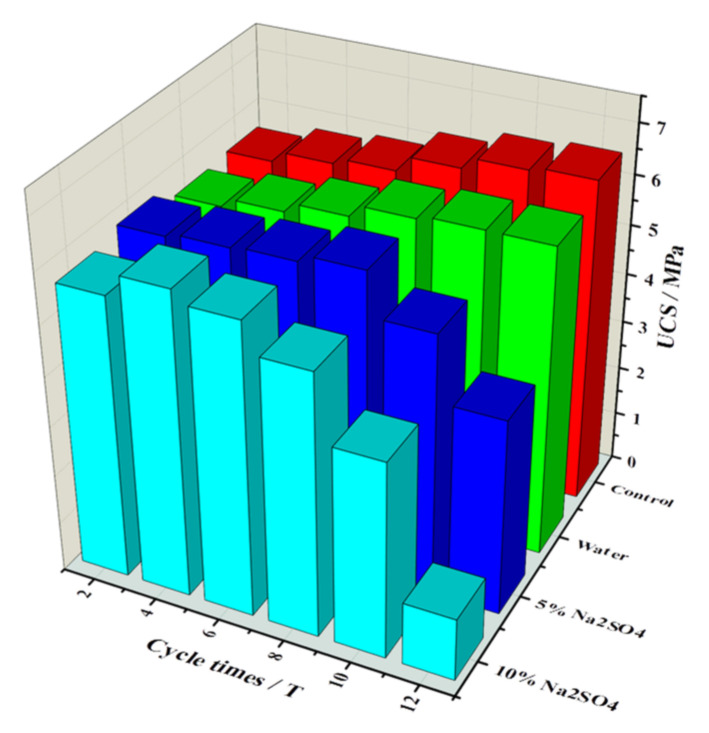
Histogram for UCS under different erosion conditions.

**Figure 9:**
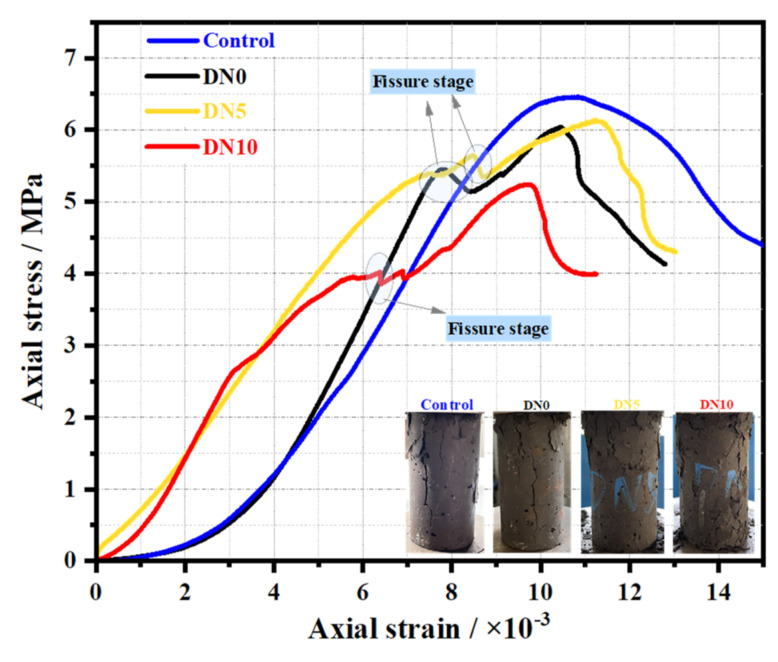
Stress–strain plots for specimens subjected to erosion with different solutions.

The average UCS was derived and a histogram was plotted based on the UCS data for different erosion conditions, as shown in Figure 8. The “Control” in the figure represents a standard conditioned specimen that has not been eroded. After the eighth cycle, the UCS values for specimens experiencing erosion with the Na_2_SO_4_ solutions were all smaller than the UCS values seen after erosion with water. Specimens from the eighth cycle were selected for this testing, and one specimen in each group was selected for stress–strain analysis. The stress–strain differences between specimens were compared for different concentrations of erosion solution at the same cycle, as shown in Figure 9.

Figure 8 shows that the UCS values of specimens attacked by the 10% Na_2_SO_4_ solution were greatest after four cycles, with a value of 6.104 MPa. In comparison, the UCS for specimens attacked by the 5% Na_2_SO_4_ solution increased at a slightly smaller rate and reached its greatest value, 6.102 MPa, after eight cycles. The UCS values of specimens eroded by Na_2_SO_4_ solutions were lower than those of specimens eroded by water after all 8–12 cycles of the UCS test. The greater the concentration of the solution was, the deeper the erosion and the lower the UCS. The strength loss rate with the 5% Na_2_SO_4_ solution erosion was approximately 40% after 12 dry–wet cycles; the strength loss rate for erosion with the 10% Na_2_SO_4_ solution was more than 80%.

Figure 9 shows that specimens subjected to erosion exhibited lower peak stresses in the UCS tests after dry–wet cycling under different conditions compared to uneroded specimens. The peak strength stresses were relatively low for specimens subjected to erosion with 10% Na_2_SO_4_. The internal pores of specimens eroded with Na_2_SO_4_ may have been filled due to the formation of erosion products, which would have resulted in premature stress damage. In addition, in the stress–strain curve shown in Figure 9, the phenomenon of fluctuations before the peak stress was the “Fissure stage”. It can be seen that the specimens eroded by the high sulphate concentrations cracked first. This indicates a release of strain energy, followed by compaction of the specimen, and finally the peak stress, at which point the specimen was damaged. This result was similar to those obtained in a study of fly ash cementitious materials by Cui et al [20]. It is worth noting that the specimens under different erosion conditions exhibited different fluctuations at the “Fissure stage”, with the fluctuations decreased in the order of DN0 > DN5 > DN10. This phenomenon of delayed damage occurred during the plastic deformation phase of the stress–strain curve. At this point, the appearance of microcracks in the specimen and the increase in the number of cracks with increasing stress indicated that damage to the specimen had begun [20]. It can be seen that higher concentrations of sulphate accelerate the erosion of the specimen and reduce the strength of the SWCPB, thus reducing the ability of the specimen to resist stress damage. Observations of the damaged specimens showed that the depth of erosion increased with increasing sulphate concentration. During the tests, damage debris continued to accumulate around the core of the specimen and residual stresses existed in all specimens after the peak stress.

### 3.3. Analysis of Erosion Products

In conjunction with the macroscopic properties of SWCPB undergoing sulphate attack, this section presents microscopic analyses the erosion products within the SWCPB material with XRD and SEM. The process for degradation due to microscopic damage to SWCPB materials caused by dry–wet cycling and sulphate attack was investigated, and the damage mechanism was revealed.

#### 3.3.1. XRD Analysis

**Figure 10:**
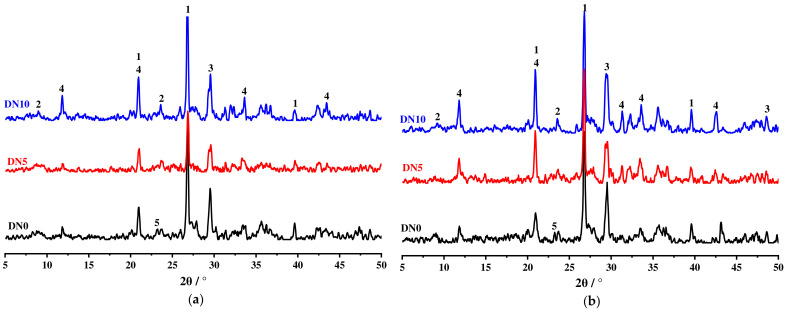
XRD diagrams for the erosion products: (**a**) XRD for erosion products from different solutions after the 8th cycle; (**b**) XRD for erosion products from different solutions after the 12th cycle. (1: quartz (SiO_2_); 2: ettringite (AFt); 3: calcite (CaCO_3_); 4: gypsum (CaSO_4_·2H_2_O); 5: calcium hydroxide (Ca(OH)_2_).)

Erosion products were generated for different cycles of erosion. The products were analysed using X-ray diffraction techniques, as shown in Figure 10.

As shown in Figure 10, the specimens exhibited distinct diffraction peaks in the XRD pattern after erosion by dry–wet cycling. According to their corresponding diffraction angles, these diffraction peaks corresponded to quartz (SiO_2_ PDF#85-0798), ettringite (AFt PDF#41-0217), calcite (CaCO_3_ PDF#72-1650), gypsum (CaSO_4_·2H_2_O PDF#74-1904) and calcium hydroxide (Ca(OH)_2_ PDF#50-0008). The quartz indicated by the XRD pattern was an intrinsic component of the raw material. The calcite was probably due to carbonisation produced by dry–wet cycles of the tests.

According to Figure 10a, the AFt diffraction peak in the XRD for specimen DN0 eroded in water was weak and there was no obvious diffraction peak for gypsum, but the diffraction peak for Ca(OH)_2_ was present. Diffraction peaks for AFt were present in the XRD for DN5 and DN10 and gradually increased with increasing Na_2_SO_4_ concentration, and the diffraction peak for gypsum was present in the XRD for DN10. This indicates that Ca(OH)_2_ was largely consumed after the eighth cycle, and this contributed to gradual increases in the production of AFt and gypsum as the concentration of the eroding solution was increased. A comparison of Figure 10a,b shows that the XRD peak shape was sharper after 12 erosion cycles than after 8 cycles. This indicates that crystallisation of the erosion products gradually stabilised and more crystallised material formed as the cycling period was increased.

In agreement with findings in the literature [26], gypsum expanded more than the AFt produced by the hydration reaction. The growth of gypsum and AFt led to gradual internal cracking and pitting [27]. This provided a larger surface area for chemical reactions to take place. The combined analysis suggests that AFt and gypsum are the main erosion products.

#### 3.3.2. SEM–EDS Analysis

The XRD analysis showed that the main products of erosion were AFt and gypsum. To further explore the morphology of the chemical products produced in CPB under different conditions, the CPB specimens were analysed with EDS, and the morphology of the chemical products was observed with SEM.

**Figure 11:**
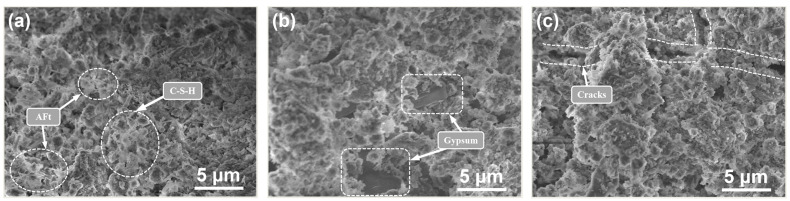
SEM showing erosion produced after the 8th cycle with different concentrations: (**a**) water (DN0); (**b**) 5% Na_2_SO_4_ solution (DN5); (**c**) 10% Na_2_SO_4_ solution (DN10).

**Figure 12:**
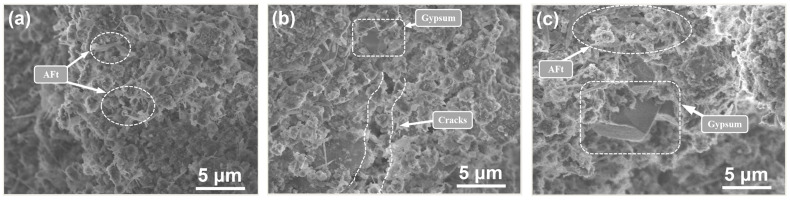
SEM showing erosion produced after the 12th cycle with different concentrations: (**a**) water (DN0); (**b**) 5% Na_2_SO_4_ solution (DN5); (**c**) 10% Na_2_SO_4_ solution (DN10).

In response to data for the aforementioned UCS losses and mass changes, this section describes SEM tests conducted on specimens after the 8th and 12th dry–wet cycles; the results are shown in Figure 11 and Figure 12, respectively.

**Figure 13:**
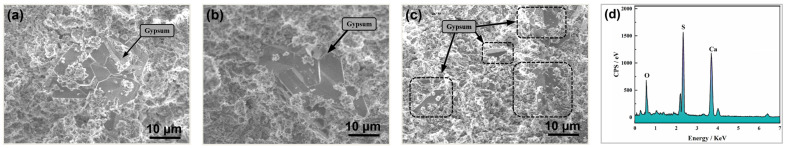
Different distribution patterns and EDS energy spectra of gypsum: (**a**–**c**) distribution patterns; (**d**) EDS energy spectrum of gypsum.

Figure 11 shows the erosion produced after the eighth cycle (mid-erosion). In Figure 11a, AFt and calcium silicate hydrate (C–S–H) gels are indicated by the SEM for DN0 eroded by water. In contrast, crystals with prismatic and plate-like morphologies were found embedded in sample DN5 after erosion with the Na_2_SO_4_ solution, and the crystals were confirmed to be gypsum after the analysis of the constituent elements with EDS (Figure 13d). In addition, cracks were found in DN10. A comprehensive analysis was carried out by combining macroscopic experiments. It was effectively confirmed that the extent of erosion damage increased when the concentration of Na_2_SO_4_ was higher, and cracks were observed on the surfaces of the specimens.

The analyses shown in Figure 11 were combined with those in Figure 12. For DN5, Figure 12b shows larger cracks caused by erosion of the gypsum compared to those shown in Figure 11b. In Figure 12c, even larger gypsum crystals are observed. This indicated that the size in erosion products gradually increased and that the degree of erosion gradually deepened with additional erosion cycles.

The SEM images shown in Figure 11a and Figure 12a are additionally noteworthy. Compared to that in Figure 3b, as the number of cycles was increased, the amount of formed needle-like AFt decreased, the volume increased, and most of the specimens were gel-like materials. It is possible that the dry–wet cycles influenced the growth of AFt. After the specimens were wetted in solution, they were transferred to a drying oven at 80 °C for drying. This high drying temperature may have inhibited the growth of AFt [28]. When the specimen was dried and re-entered a humid environment, hydration re-generated AFt. This causes the interior of a specimen to develop a phenomenon called delayed ettringite formation (DEF) at the pores, which increased the volume of the solids in the pores and damaged the CPB specimen.

Figure 13 shows different distribution patterns in the pores of the SWCPB for erosion products (gypsum) formed during erosion by the Na_2_SO_4_ solution, as well as their EDS energy spectra.

### 3.4. Analysis of the Erosion Mechanism

**Figure 14:**
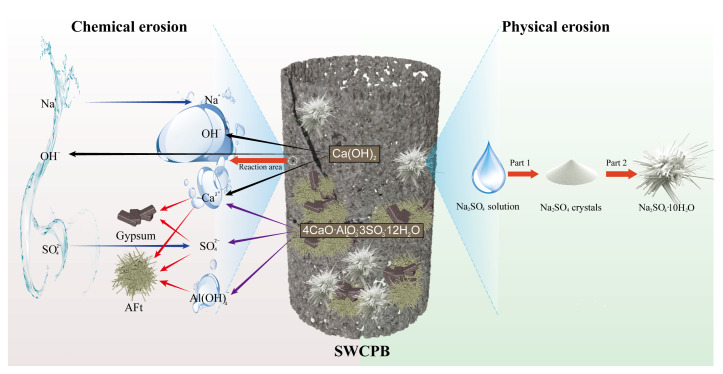
Schematic diagrams of the erosion mechanism.

The experimental phenomena and conclusions obtained from the abovementioned experiments were collated and summarised, a comprehensive analysis of the erosion resistance of SWCPB prepared from OSR–SS–SR was undertaken, and the mechanism for erosion of this CPB material by dry–wet cycling with sulphate solutions was analysed. The erosion process was divided into two parts: physical erosion and chemical erosion. The main schematic diagrams are shown in Figure 14.

#### 3.4.1. Physical Erosion

As shown in Figure 14, the physical erosion of SWCPB materials by sodium sulphate was divided into two main processes.

Part 1: During the dry–wet cycles, water evaporation from within the SWCPB during the dry periods led to increases in the solution concentration and, consequently, the crystallisation of Na_2_SO_4_. The white crystals observed on the surface of the specimen during the experiments described in Section 3.2.1 were produced by this process.

Part 2: As the specimen transformed from the dry state to the wet state, internal crystals of anhydrous Na_2_SO_4_ were converted to crystals of the hydrate Na_2_SO_4_·10H_2_O. During this process, the crystals increased in volume by 315% [26]. The chemical reaction is shown in Equation (7).


(7) $${\mathrm{Na}}_{2}{\mathrm{SO}}_{4}+{10H}_{2}O\to {\mathrm{Na}}_{2}{\mathrm{SO}}_{4}\cdot 10H_{2}O$$

Other scholars [29,30] studied the crystallisation of Na_2_SO_4_ with environmental scanning electron microscopy (ESEM). They found that anhydrous sodium sulphate dissolved during the transformation to hydrated sodium sulphate and then crystallised. It was not the absorption of waters of crystallisation that directly produced the volume expansion. However, cycling between dry and wet cycles in the experiment resulted in large variations in temperature and humidity in the environment of the specimen. This then led to the crystallisation and production of Na_2_SO_4_·10H_2_O or Na_2_SO_4_ crystals. In summary, the mechanism for physical erosion involved the destruction of crystalline Na_2_SO_4_.

#### 3.4.2. Chemical Erosion

Chemical attack was found to be mainly related to the concentration of Na_2_SO_4_. When the ${\mathrm{SO}}_{4}^{2-}$ concentration was low, AFt was mainly produced, and high concentrations of ${\mathrm{SO}}_{4}^{2-}$ led to the formation of gypsum crystals.

1. Formation of Aft.

As shown in Equation (3), the AFt generated by the raw material itself allowed it to develop strength during the curing period. However, when the SWCPB was subjected to erosion by the Na_2_SO_4_ solution, ${\mathrm{SO}}_{4}^{2-}$ reacted with the remaining Ca(OH)_2_ to produce more AFt. This caused the SWCPB to increase in mass and generate a larger UCS during short-term erosion. As the number of erosion cycles increased, the excess AFt led to an increase in solid volume, which caused swelling and cracking. The reaction is shown in Equation (8):


(8) $$3\mathrm{CaO}\cdot {3\mathrm{Al}}_{2}O_{3}\cdot {\mathrm{CaSO}}_{4}+{8\mathrm{CaSO}}_{4}+6\mathrm{CaO}+96H_{2}O\to 3(3\mathrm{CaO}\cdot {\mathrm{Al}}_{2}O_{3}\cdot {3\mathrm{CaSO}}_{4}\cdot 32H_{2}O)$$

2. Formation of Gypsum.

When the Na_2_SO_4_ concentration was high, the formation of AFt was accompanied by the precipitation of gypsum. On the one hand, the solid volume increased by 124% when gypsum was formed, which caused the SWCPB to crack [21,31]. On the other hand, the production of gypsum consumed Ca(OH)_2_ and reduced the alkalinity within the specimen; this resulted in the decomposition of hydration products such as C–S–H, which led to a loss of strength and a reduction in the durability of the SWCPB. The production of gypsum is demonstrated in Equations (9) and (10):


(9) $${\mathrm{Na}}_{2}{\mathrm{SO}}_{4}+\mathrm{Ca}{\left(\mathrm{OH}\right)}_{2}\to {\mathrm{Ca}}^{2+}+{\mathrm{SO}}_{4}^{2-}+{\mathrm{Na}}^{+}+{\mathrm{OH}}^{-}$$

(10) $${\mathrm{Ca}}^{2+}+{\mathrm{SO}}_{4}^{2-}+2H_{2}O\to {\mathrm{CaSO}}_{4}\cdot 2H_{2}O$$

In summary, the same factors as conventional cementitious materials exist for SWCPB. The chemical processes occurring during sulphate erosion are all related to Ca(OH)_2_ content in the material [32]. The differences lie in the sources and contents of Ca(OH)_2_. Ca(OH)_2_ in conventional CPB is mainly derived from cement and is present in large amounts. Many scholars have proposed that an effective reduction in Ca(OH)_2_ content in a material would improve the resistance of the material to sulphate attack [19,32]. The Ca(OH)_2_ in SWCPB is mainly derived from SS. SWCPB uses mutual interactions between solid wastes to produce cementation strength. The Ca(OH)_2_ present in the SS and the SR work together to create an alkaline environment that promotes the continuous hydration of SiO_2_ and Al_2_O_3_ in the OSR. As a result, the hydration reaction consumes more Ca(OH)_2_ from the SS. This also makes it more resistant to sulphate attack. The most significant cause of erosion is the production of gypsum. As its volume expands and cracks form, it promotes the rate at which ions in solution enter the material, which in turn increases the contact area with the material. Therefore, more erosion products (gypsum and AFt) are generated, and they are accompanied by the crystallisation of Na_2_SO_4_, which together lead to erosion damage to SWCPB.

## 4. Conclusions

SWCPB prepared from OSR–SS–SR was tested with dry–wet cycles and erosion by sodium sulphate solutions. The evolution of damage to the SWCPB in a complex filling environment was summarised, and the mechanism for the deterioration of the material was revealed. The conclusions are as follows:

(1) The combined effects of SWCPB erosion by Na_2_SO_4_ and dry–wet cycles develop in pores in contact with the SWCPB. These results are microscopically characterised by a continuous build-up of erosion products and the destructive effects of Na_2_SO_4_ crystal expansion. This produces microcracks in the erosion zone of the SWCPB. These microcracks are continuously expanded and connected, which eventually leads to the cracking and destruction of the SWCPB; this is a continuous process.
(2) The erosion products formed in the SWCPB eroded by high concentrations of Na_2_SO_4_ are mainly AFt and gypsum. Due to the production of DEF, gypsum becomes the main erosion product from the SWCPB. This improves the performance of the material in the early stages of erosion.
(3) The stress–strain curves show that specimens subjected to high concentrations of sulphate were the first to show cracking. These eroded specimens all showed delayed damage, and the delayed damage caused by water erosion was significantly greater than that caused by sulphate solution erosion.
(4) SWCPB makes full use of synergistic effects involving raw materials, and the hydration reaction consumes Ca(OH)_2_ within the material and provides some resistance to sulphate erosion. This indicates that the characteristics of components in the solid waste enable the material to meet filling requirements and also provide resistance to erosion.
(5) This paper is focused on filling environments with high concentrations of sulphate in mine water. In the future, the influence of different filling environments on SWCPB should be further investigated. SWCPB is a candidate material for filling in the mining process, and filling field experiments and tests are still needed to promote its use. Cores were drilled and samples made from paste in the field filling area were used to compare durability properties with those of specimens in the laboratory.

## Figures and Tables

**Table 1:** Main chemical compositions of the raw materials (wt%).

Material	Al_2_O_3_	CaO	SiO_2_	Fe_2_O_3_	MgO	SO_3_	Na_2_O	TiO_2_	K_2_O
OSR	14.71	7.15	62.16	8.32	2.38	0.82	0.04	0.87	3.07
SS	6.15	40.75	12.39	14.17	21.21	2.79	0.03	0.81	0.15
SR	4.31	66.12	9.68	0.29	0.31	11.21	1.99	0.22	1.32

**Table 2:** Optimum mixing ratio and test data for SWCPB.

Ratio of Solid Mass Concentration/%	Ratio of OSR in Total Solids/%	Ratio of SS In Total Solids/%	Ratio of SR in Total Solids/%	28 d UCS /MPa	Slump /mm	Cost /$
70.32	40.54	47.43	12.03	5.97	200	4.73

## Data Availability

Data can be obtained from corresponding authors upon reasonable request.
